# Supplementary material for: Scientific text citation analysis using CNN features and ensemble learning model
Source: PLoS One. 2024 May 28;19(5):e0302304. doi: 10.1371/journal.pone.0302304 (PMC11132466; doi:10.1371/journal.pone.0302304)
Supplement: Attachment — Submitted filename: Reply_to_reviewer__PONE_D_23_24549_Khaled.pdf [file pone.0302304.s001.pdf]

Manuscript ID: PONE-D-23-24549

Article title: Scientific Text Citation Analysis Using CNN Features and Ensemble Learning Model

November 15, 2023

Dear Associate Editor,

First of all, we would like to extend our sincere gratitude to Associate Editor, for allowing a re-submission of our manuscript, with an opportunity to address the reviewers'comments. We greatly appreciate the Reviewers' time spent reviewing the paper and assisting in its improvement. These comments are very helpful for revising and improving the paper, as well as the important guiding significance to the other research. We have studied the comments very carefully and made the corrections that we hope to meet with the final approval. We have done our best to modify the paper to reflect all the comments made by the Associate editor and anonymous reviewers. These changes are fully addressed in this reply letter.

Below, we provide point to point responses explaining how we have addressed each of the Reviewer's comments. For clarity, our responses have been written in blue color.

Reviewer 1

Concern # 1

The Abstract must include the statistical findings and the main contribution.

We are thankful for your feedback and appreciate your comments. Respected reviewer, We have revised the Abstract.

Concern # 2

Add more related works

Thank you for your positive feedback. We have added more works in related work.

The authors in [4] divided the citations into two categories: "influential" and "non-influential" The machine learning method (SVM) was used to help with this classification. A hundred research papers from the ACL Anthology were included in the dataset. There were five unique characteristics used: citation frequency, similarity, context, position, context, and other factors. A study [1] presented a content-based method for sentiment analysis in in-text citations, utilising binary classification to

differentiate between significant and unimportant citations. The analysis' sentiment and cosine similarity scores were used as features in binary classification algorithms, filling a gap in the state-of-the-art literature where the ideal model for sentiment analysis was unknown. The work used automated sentiment analysis on extracted in-text citation material, tested several categorization models, and improved the understanding of sentiment analysis in citation settings.

Previously, numerous methods for identifying noteworthy citations were presented, including content-based, meta-data-based, and bibliographic-based approaches. However, even though the results were cutting-edge, they still needed to be improved. Authors in [2] used a two-module technique that included section-wise citation counting and sentiment analysis of citation sentences. The first module used Neural Network and Multiple Regression for automated weight assignment, while the second module used sentiment analysis for sentence-based categorization utilising Random Forest, Support Vector Machine, and Multilayer Perceptron. Another research [3] proposed a new machine learning framework to distinguish important from non-important citations by analyzing syntactic and contextual information. Using three feature selection algorithms and three classifiers, the study identified key features for differentiation. Experimentation on two datasets demonstrated the framework's superior classification performance compared to contemporary research, highlighting the significance of both syntactic and contextual features in identifying important citations.

Concern # 3

Methodology as figure its better

Respected reviewer, We have already added figure 1 & 2 in the paper to show the complete methodology and workflow of the proposed framework. The figure are:

Reviewer 2

Concern # 1

There are some incomplete sentences and some statements do not make much sense; e.g. in the abstract, the proposed method has been claimed to 'outperformed' (line-17) but there was no specification found what models were performing less than the proposed one).

Respected reviewer, We claimed that the proposed model outperformed other machine learning models used in the paper like DT, Adaboost, LR, SGD, RF, ETC, and

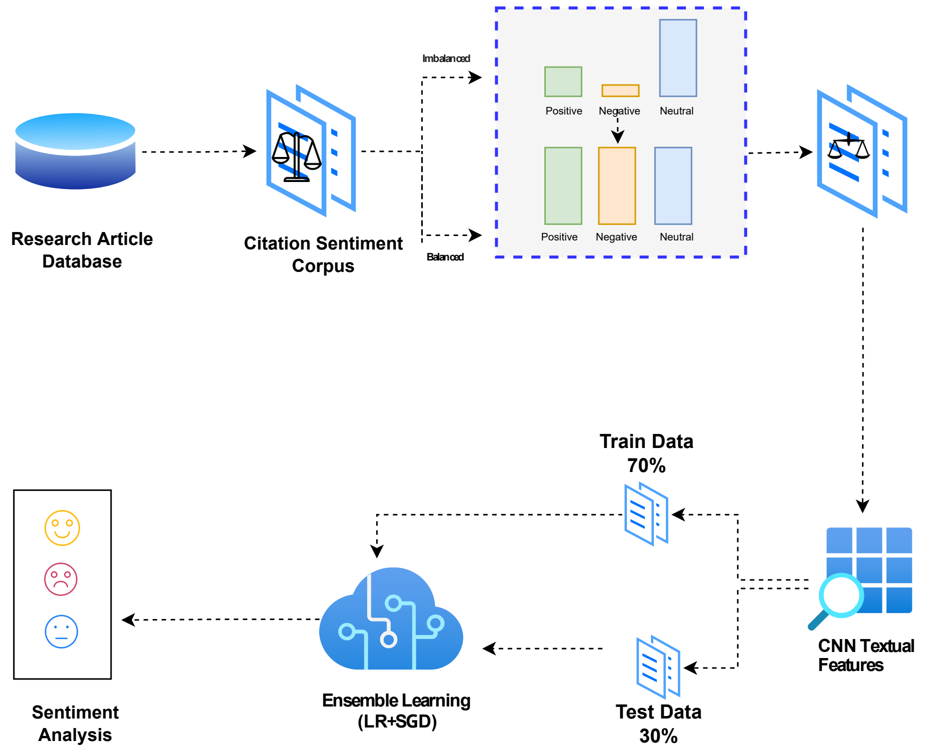

Figure 1: Proposed Architectural Diagram

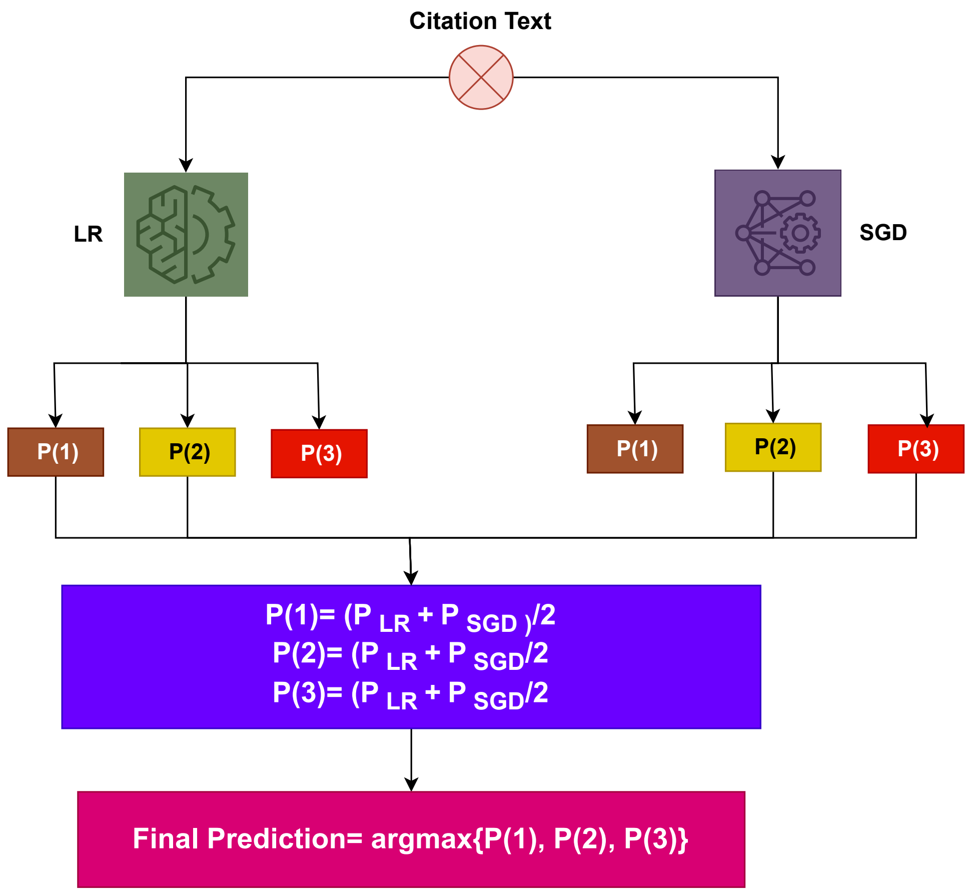

Figure 2: Architecture of the proposed voting classifier (LR+SGD) model.

SVC. For clarity, we have revised the sentence in the abstract. The updated abstract is:

Citation illustrates the link between citing and cited documents. Different aspects of achievements like the journal's impact factor, author's ranking, and peers' judgment are analyzed using citations. However, citations are given the same weight for determining these important metrics. However academics contend that not all citations can ever have equal weight. Predominantly, such rankings are based on quantitative measures and the qualitative aspect is completely ignored. For a fair evaluation, qualitative evaluation of citations is needed in addition to quantitative ones. Many existing works that use qualitative evaluation consider binary class and categorize citations as important or unimportant. This study considers multi-class tasks for citation sentiments on imbalanced data and presents a novel framework for sentiment analysis in in-text citations of research articles. In the proposed technique, features are retrieved using a convolutional neural network (CNN), and classification is performed using a voting classifier that combines Logistic Regression (LR) and Stochastic Gradient Descent (SGD). The class imbalance problem is handled by the synthetic minority oversampling technique (SMOTE). Extensive experiments are performed in comparison with the proposed approach using SMOTE-generated data and machine learning models by term frequency (TF), and term frequency-inverse document frequency (TF-IDF) to evaluate the efficacy of the proposed approach for citation analysis. It is found that the proposed voting classifier using CNN features achieves an accuracy, precision, recall, and F1 score of 0.99 for all. This work not only advances the field of sentiment analysis in academic citations but also underscores the importance of incorporating qualitative aspects in evaluating the impact and sentiments conveyed through citations.

(Concern # 2)

Two different classifiers were chosen to perform a voting classifier; this is not a standard practice; normally an odd number (e.g. 3, 5, ..) of classifiers would be chosen for a voting classifier model. No justification has been provided for choice of two classifiers (LR and SGD) for the voting classifier; how those two classifiers were selected among many others? Yet, the combination of these models has been already shown in a previous work (which has been already cited by the authors [44]), there is not much novelty found in the work presented in this paper.

We are thankful for your feedback and appreciate your comment regarding the use of two classifiers (LR and SGD) for the voting classifier. We appreciate the insightful observation and would like to provide a clarification on this choice. The decision to

employ Logistic Regression (LR) and Stochastic Gradient Descent (SGD) as the two classifiers for the voting classifier is not luck by chance. As you can when we use CNN features with machine learning models than top 3 classifiers are LR, SGD, and RF. We also applied voting ensemble of LR+RF, and SGD+RF but results are not much considerable and less than the voting ensemble of LR+SGD. The reason for betterment of LR+SGD lies in their way of handling sparse data as in text mining, CNN features are not so much related to each other. For further clarification we have added the results of LR+RF and SGD+RF in the table 7 of the paper.
Furthermore, We acknowledge that our model selection aligns with the existing literature, as demonstrated in a cited work [44] but the theme of both papers is completely different. Cited work [44] makes uses of four word embedding techniques fastText, fastText subword, GloVe and fusion of these 3 with CNN as proposed model. While in our research work, CNN is used as a new feature engineering techniques and voting classifier is the proposed model that clearly outperforms all the experimental results of cited work [44]. That is why we also make a complete results comparison with all approaches used in the cited work [44] and many others.

Concern # 3

The total sample size was given (line 158) however exact sample size for each class was not explicitly provided; similarly the readers are given the training:testing split ratio of 70%:30% but we do not know how many samples in each class and in both training and testing set exist. Similarly, after data augmentation (i.e. using SMOTE), the number of the samples exist in each class was not specified.

Respected reviewer, we have updated the dataset section and provided complete details about the dataset. The updated text is:

The dataset contains 8,736 citation text annotated by human experts. Out of 8,736 citations records 829 are positive, 280 negative, and 7627 are neutral. These 8,736 citations are extracted from 194 research articles. After SMOTE augmentation/upsampling, we have 1000 citation records of each class to balance the dataset. The dataset comprises 'Source_Paper ID', 'Target_Paper ID', 'Citation_Text', and 'Sentiment'. The 'Source_Paper ID' is the citing paper's ID that represents the source of the text, 'Target_Paper ID' is the cited paper's ID, 'Citation_Text' is the original text that contains the citation while 'Sentiment' is the label of the target class and can be 'positive', 'negative', or 'neutral'.

Concern # 4

No discussion provided about why the voting classifier was one of the worst performing

ones when the SMOTE was employed to balance the classes' sample sizes (e.g. Tables 4 and 6).

We are thankful for your feedback and appreciate your comments. We have provided the discussion. Several factors could contribute to this decrease in performance. First, the synthetic samples generated by SMOTE may introduce noise or artificial patterns that do not align well with the true distribution of the data. This can adversely impact the decision boundaries learned by the voting classifier, resulting in a decrease in overall performance. Second, the performance of VC is poor in this case because both classifiers have the same way of learning feature patterns (linear feature capturing). If the features are quite similar to each other, than there are more chances that both classifiers makes similar types of errors and performs poor like in our case. We have also added these reasons to the paper.

Concern # 5

No comments provided on why TF and TF-IDF use have been concluded with the exact same performance values for all the classifiers (in both cases when SMOTE is employed and not employed)?

We are thankful for your feedback and appreciate your comments. Respected reviewer, This consistency in performance across TF and TF-IDF scenarios may arise from several factors like the specific characteristics of the dataset and the nature of the text features extracted by TF and TF-IDF might not significantly differentiate between the two methods in the context of sentiment analysis on the given data. Furthermore, the choice of classifiers and their hyperparameter settings could contribute to this uniformity in performance. If the classifiers are robust and well-tuned for the given task, variations introduced by different feature extraction methods (TF and TF-IDF) might not significantly impact their performance.

Concern # 6

The bars in Fig-3 that represent the accuracy of the VC do not look quite correct (especially the final 2 bars representing TF+CNN features and TF-IDF+CNN features) according to the text and tables provided in the manuscript

We appreciate your insightful comments. Respected reviewer, we have added updated Figure 3 of the manuscript as shown in Figure 3

Concern # 7

There is not any novelty in this draft when compared to the cited work [44] (https://doi.org/10

Respected reviewer, We acknowledge that our model selection aligns with the existing literature, as demonstrated in a cited work [44] but the theme of both papers is completely different. Cited work [44] makes uses of four word embedding techniques fastText, fastText subword, GloVe and fusion of these 3 with CNN as proposed model. While in our research work, CNN is used as a new feature engineering techniques and voting classifier is the proposed model that clearly outperforms all the experimental results of cited work [44]. That is why we also make a complete results comparison

with all approaches used in the cited work [44] and many others.

Concern # 8

The results shown in the Tables 3 to 7 were expected to be shown on the test set only (the results for the training could have been given as a suppl data or could have been provided explicitly in the main document) but the correct comparison between the models should be performed on the test set. As a follow up comment; we do not know which data set's (training or testing) performance results have been shown in the tables 3 to 7 and how and why the results in Table-4&6 differ than the ones shown in Table-9.

We are thankful for your insightful comments. Respected reviewer, It was a typo error, we have corrected it.

Reviewer 3

Concern # 1

The scheduler works in a hierarchical way as shown in Figure 1&2 and Algorithm 1. Jobs coming from multiple queues are split into tasks and sent to master schedulers. While reasonably written, presentation has some problems as it shows multiple algorithms with no clear relation between them. For example, is algorithm 4 ever referred to and described in the text?

Respected reviewer, We appreciate the time and effort you have invested in reviewing our work. We have carefully considered your concern about the presentation, specifically regarding the apparent lack of a clear relation between multiple algorithms

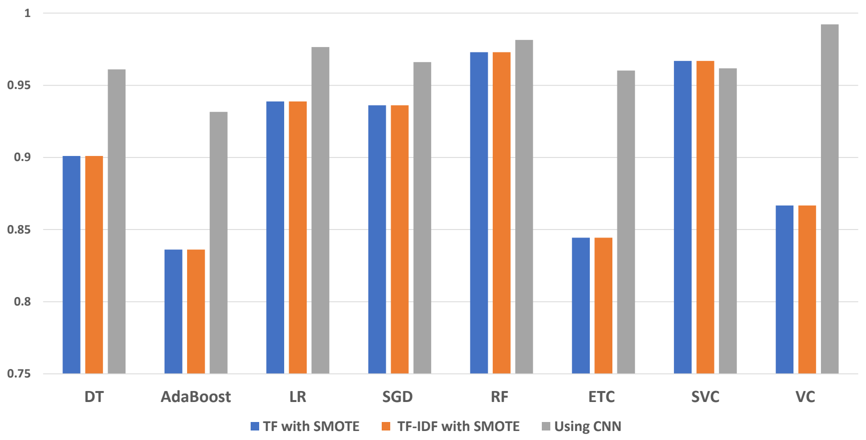

Figure 3: Accuracy Comparison of Classifiers

and the absence of a reference to Algorithm 4 in the text. we would like to clarify that there is no Algorithm 4 in the paper. It seems there may be a misunderstanding, and we apologize for any confusion.

(Concern # 2)

None of the ideas in this paper is new, e.g., as mentioned by author in references.

Respected reviewer, We acknowledge that our model selection aligns with the existing literature, as demonstrated in a cited work [44] but the theme of both papers is completely different. Cited work [44] makes uses of four word embedding techniques fastText, fastText subword, GloVe and fusion of these 3 with CNN as proposed model. In our research work, CNN is used as a new feature engineering technique and the voting classifier is the proposed model that clearly outperforms all the experimental results of the cited work [44]. That is why we also make a complete results comparison with all approaches used in the cited work [44] and many others.

Concern # 3

I believe that it is fair to say that authors built a variation of previously existing ideas and tried them in a fairly complex experimental setting. This is not to say that the implementation does not have anything new and different.

Respected reviewer, We appreciate the reviewer's acknowledgement of the experimental complexity and the recognition that our work involves a variation of previously existing ideas. While the core ideas might share a foundation with existing work, our implementation introduces innovative aspects, such as the combination of specific classifiers within the voting classifier, the use of SMOTE to address the class imbalance and the incorporation of TF and TF-IDF for feature extraction. These modifications are designed to enhance the performance of sentiment analysis in academic citations.

Concern # 4

Graphics depicted are somewhat limited and straightforward, as only a handful of algorithms were used, and they are not necessarily aligned with the proposed scheduler in their goals.

Respected Reviewer, The selection of algorithms presented in the graphics was intentionally limited to those that highlight key aspects of the proposed model's functionality. Our aim was to offer a clear and focused representation that emphasizes the core principles and features of our proposed approach. We have added figure 1 & 2 in the paper to show the complete methodology and workflow of the proposed

framework.

Concern # 5

Either Bracket (or) are missing/abused in many places, such as in equation (6), figure 2.

We are thankful for your feedback and appreciate your comments. We have added Brackets in the above-mentioned places.

References

[1] Hanan Aljuaid, Rimsha Iftikhar, Shahbaz Ahmad, Muhammad Asif, and Muhammad Tanvir Afzal. Important citation identification using sentiment analysis of in-text citations. *Telematics and Informatics*, 56:101492, 2021.

[2] Shahzad Nazir, Muhammad Asif, Shahbaz Ahmad, Hanan Aljuaid, Rimsha Iftikhar, Zubair Nawaz, and Yazeed Yasin Ghadi. Important citation identification by exploding the sentiment analysis and section-wise in-text citation weights. *IEEE Access*, 10:87990–88000, 2022.

[3] Mingyang Wang, Jiaqi Zhang, Shijia Jiao, Xiangrong Zhang, Na Zhu, and Guangsheng Chen. Important citation identification by exploiting the syntactic and contextual information of citations. *Scientometrics*, 125:2109–2129, 2020.

[4] Xiaodan Zhu, Peter Turney, Daniel Lemire, and André Vellino. Measuring academic influence: Not all citations are equal. *Journal of the Association for Information Science and Technology*, 66(2):408–427, 2015.
