# Supplementary material for: Scientific text citation analysis using CNN features and ensemble learning model
Source: PLoS One. 2024 May 28;19(5):e0302304. doi: 10.1371/journal.pone.0302304 (PMC11132466; doi:10.1371/journal.pone.0302304)
Supplement: Attachment — Submitted filename: R2_Reply_to_reviewer__PONE_D_23_24549_Khaled.pdf [file pone.0302304.s002.pdf]

Manuscript ID: PONE-D-23-24549

Article title: Scientific Text Citation Analysis Using CNN Features and Ensemble Learning Model

January 20, 2024

Dear Associate Editor,

First of all, we would like to extend our sincere gratitude to Associate Editor, for allowing a re-submission of our manuscript, with an opportunity to address the reviewers'comments. We greatly appreciate the Reviewers' time spent reviewing the paper and assisting in its improvement. These comments are very helpful for revising and improving the paper, as well as the important guiding significance to the other research. We have studied the comments very carefully and made the corrections that we hope to meet with the final approval. We have done our best to modify the paper to reflect all the comments made by the Associate editor and anonymous reviewers. These changes are fully addressed in this reply letter.

Below, we provide point to point responses explaining how we have addressed each of the Reviewer's comments. For clarity, our responses have been written in blue color.

Reviewer 1

Concern # 1

All of my comments are addressed.

I am very thankful for your feedback and appreciate your comments that improves the paper quality to an acceptable form.

Reviewer 2

Concern # 1

Novelty of this paper is not clear to me, anything has been implemented in the paper was already found on literature (eg a combination use of SMOTE with some ML algorithms or feature extraction with CNN despite in [44] a different set of embedding has been used, they still use the CNN concept for feature extraction..)

Respected reviewer, reference [44] is "IoT Based Smart Monitoring of Patients with Acute Heart Failure" which explores the use of machine learning models, such as CNN,

for heart failure prediction in a smart healthcare framework based on 13 categorical and continuous features. However, we would like to highlight the distinctive contributions and innovations in our work. we emphasize the distinctive contributions of our research, which focuses on sentiment analysis in in-text citations, an unexplored area. Unlike existing studies, we introduce a multi-class sentiment analysis approach and employ a unique combination of techniques, including a convolutional neural network (CNN) for feature extraction and a synthetic minority oversampling technique (SMOTE) for handling imbalanced data. These aspects collectively distinguish our work and contribute to the field of sentiment analysis in academic citations.

(Concern # 2)

Logistic Regression and SVM are binary classifiers, methods section could have explained how they were covered to a multi class classifiers (eg one-vs-one, one-vs-all; which has been described only for SGC but not for LR or SVM)

I am thankful for your feedback. I have added details for LR and SVM model. The details are alike:

LR is basically designed for binary classification but in this research, I have used LR One-vs-All (OvA) technique for classification. For each class, a binary classification model is trained to distinguish that class from all other classes. The final prediction is then based on the class with the highest probability among the individual classifiers.

SVC is basically designed for binary classification but in this research like LR, I have used SVC as One-vs-All (OvA) or One-vs-Rest (OVR) technique with linear kernel (OneVsRestClassifier(SVC(kernel='linear'))) for classification. For each class, a binary classification model is trained to distinguish that class from all other classes. The final prediction is then based on the class with the highest probability among the individual classifiers.

Concern # 3

No other implementation details provided on the materials (parameter or hyperparameter settings eg number of trees in RF, kernel type and values used in SVC etc)

Respected reviewer, I have provided all these details in the paper. The details are: Table 1 provides implementation details about these machine learning models, and their hyperparameter settings for all of them. To find the best parameters, a method called grid search is used. This involves trying different values for each parameter within a specified range and evaluating how well the model works. Every parameter

goes through the procedure, and the values that optimize the model's performance are selected at the conclusion.

Table 1: Hyperparameter details of all machine learning models.

Classifier	Hyperparameter setting
RF	random_state=50, max_depth=20, n_estimators=200
AdaBoost	random_state=50, max_depth=20, n_estimators=200
SVC	C = 2:0, random_state=500, kernel='linear'
LR	solver='saga', C = 3:0, max_iter=100, penalty='l2'
SGD	Larning_rate='optimal', epsilon = 0.2
DT	max_depth = 25, max_depth = 2–50
ETC	random_state=50, max_depth=20, n_estimators=200
VC	Voting='hard'

Concern # 4

On the line 298 (Page 8) it should be 'convolutional layer', not 'conventional'

Respected reviewer, thank you so much for pointing out this mistake. I have corrected it and thoroughly checked this section for other typos.

Concern # 5

I do not agree with the discussion/conclusion made regarding why the VC was performing poor in the case of using SMOTE (tables 4 and 6); 'if both classifiers have the same way of learning feature patters' as claimed by the author, then their common use would perform somewhere close to the individual use of these models (eg 93% of accuracy) since they would generate similar probability scores for a given data point. Contrarily, I think these two models learn different (non-consensus) patterns for a given data point hence they agree lesser on the final probability value of VC.

We are thankful for your feedback and appreciate your comments. Respected reviewer, The decrease in performance of the voting classifier (VC) with the synthetic minority oversampling technique (SMOTE) can be attributed to factors such as inherent diversity among base classifiers, potential introduction of noise or misleading synthetic samples by SMOTE, disruption of the delicate balance in ensemble diversity, varied interactions between SMOTE and base classifiers, concerns about overfitting, and sensitivity to SMOTE parameters. Addressing these issues requires further investigation into the interplay between SMOTE and specific base classifiers, experimentation with alternative oversampling techniques, and fine-tuning of SMOTE parameters for optimal performance in handling imbalanced data within the voting ensemble.

Reviewer 3

Concern # 1

Figure 1 is not very clear in the printed version. The font size (e.g., the font size of some text in the figure) is a little small, which makes it a little difficult for the readers to clearly see the content in the figure. Considering that there is enough space, it would be better to enlarge the font size a little.

Respected reviewer, I have converted figure to .eps format and submit it as separate file for review.

(Concern # 2)

The presentation of the paper can be further improved. There are some typos in the paper. Here are some examples:
a. Page 6, line 235: "tree" could be changed to "Tree" for consistency.
b. Page 6, line 238: a space between "." and "ETC" is missing.
c. Page 9, line 326: "figure 1" could be changed to "Figure 1".

Respected reviewer, bundle of thanks for pointing out these mistakes that improve the quality of the paper. I have corrected all these mistakes and cross-checked the complete paper for mistakes like this.

Concern # 3

The format of the reference could be improved. For example, the format of references 2, 3, 8, 11 is a little different from that of other references. There are two punctuations ";" and "." after the last word in the sentence.

Respected reviewer, the semi-colon appears when information like page range or volume is missing. I have provided the required information and now all references are in the same format.

Concern # 4

This paper uses the SMOTE algorithm to handle class imbalance problem. Some other related works also use SMOTE algorithm to balance the dataset (e.g., the work focusing on extracting textual features of financial social media). Many feature selection methods are usually based on one chosen approach only without considering combining dissimilar feature selection approaches to enhance the performance. Sometimes hybrid feature selection can better enhance the performance. Probably the authors can consider it in the future work.

Respected Reviewer, your point is valid, but if I do SMOTE, voting ensemble learning, and hybrid feature all things in one paper, the paper becomes too lengthy. I

added your point as future work direction at the end of the conclusion section. The added line is:

The second future work direction is to make use of hybrid feature selection like merging two different word embedding with PCA.

References
